# Multimorbidity status and annual healthcare expenditures of rheumatoid arthritis patients: a Dutch hospital-centered versus population-based comparison

**DOI:** 10.1007/s00296-023-05282-w

**Published:** 2023-02-10

**Authors:** Fiona Koster, Pieter L. H. Bakx, Marc R. Kok, Deirisa Lopes Barreto, Angelique E. A. M. Weel-Koenders

**Affiliations:** 1 Department of Rheumatology and Clinical Immunology, Maasstad Hospital Rotterdam, Rotterdam, The Netherlands; 2 Erasmus School of Health Policy and Management, Erasmus University Rotterdam, Rotterdam, The Netherlands

**Keywords:** Rheumatoid arthritis, Multimorbidity, Health expenditures, Hospital costs, Health services research

## Abstract

The prevalence of multimorbidity among rheumatoid arthritis (RA) patients is increasing and associated with worse outcomes. Therefore, management of multimorbid patients requires a multidisciplinary approach. However, healthcare systems consist of mono-disciplinary subsystems, which limits collaboration across subsystems. To study the importance of a multidisciplinary, integrated approach, associations between expenditures and multimorbidity are assessed in real-life data. Retrospective data on RA patients from a Dutch single-hospital are analyzed and compared to the Dutch RA population data. The Elixhauser index is used to measure the multimorbidity prevalence. Regression analyses were conducted to derive the relationship between multimorbidity, healthcare costs and self-reported quality of life (e.g. EQ-5D). When analyzing the impact of multimorbidity within RA patients in context of a single-hospital context, multimorbidity is only partially captured: 13% prevalence versus 24% of the Dutch population. Multimorbidity is associated with higher care expenditures. Depending on the type of multimorbidity, expenditures are €43–€5821 higher in a single-hospital and from €2259–€9648 in population data. Finally, medication use associated with chronic diseases and self-reported aspects of well-being are associated with similar increases in healthcare expenditures as multimorbidity based on hospital care. Within RA, a single-hospital approach underestimates the association between multimorbidity and healthcare expenditures as 43% of healthcare utilization and expenditures are missed. To overcome a single-provider perspective in healthcare and efficiently coordinate multimorbid patients, besides providing holistic care, professionals also need to use data providing comprehensive pictures of patients.

**Supplementary Information:**

The online version contains supplementary material available at 10.1007/s00296-023-05282-w.

## Introduction

Multimorbidity is frequently present at the onset of rheumatoid arthritis (RA) and the prevalence increases from 38 to 56.5% after 10 years [1]. Multimorbidity requires a different approach concerning the care delivery process in patients with RA as the clinical outcomes and response on treatments might be delayed or poor [2–4]. Multimorbidity is defined as the coexistence of at least two chronic illnesses in one patient, implying that RA patients with at least one other chronic disease, are multimorbid [5]. Multimorbidity is associated with lower survival, quality of life and affects treatment and therefore requires extensive and ongoing care involving a multi-disciplinary team of providers is presumably required [2]. Moreover, the available knowledge predominantly comes from investigations on multimorbidity within solely primary care or hospital (out- and inpatient) data. As a consequence, insight regarding the impact of multimorbidity on the full spectrum of healthcare costs including primary- and mental care is lacking.

The current fragmented structure of the healthcare system with facilities and departments acting as independent providers of care, impedes the integrated delivery of care for multimorbid patients. Instead, these independent providers each focus on treating single illnesses [6]. This single-illness approach causes a lack of collaboration and coordination across care settings and healthcare providers and thus may lead to errors, increased expenditures and worse health outcomes [6]. Additionally, physicians are primarily conscious of the frequently occurring associated morbidities within their disease area. By contrast, value-based healthcare (VBHC) pushes for patient-centered integrated care delivery, i.e. a holistic multidisciplinary approach. At present, a generic outcome set for adults has been established as well as a Dutch guideline including a decision aid for healthcare providers [7]. However, these outcome sets have not yet been linked to healthcare costs.

Insight in health resource use and costs of RA patients with multimorbidity helps to highlight the proper scope for multimorbid disease management through integrated care delivery. Prior prospective cohort research showed multimorbidity in RA patients is associated with increased healthcare use, higher expenditures and reduced work-related productivity [6–8]. A Scottish study showed that the annual costs significantly differed in patients with only RA compared to patients with RA and a single comorbidity [9]. In addition to expenditures, multimorbidity also impacts the quality of life of patients suffering from RA. A higher number of multimorbidities is associated with declining Euro Quality of Lifel-5 Dimension (EQ-5D) scores [10].

The goal of VBHC is to compete on the value added from a patient perspective [11]. This evaluation of costs and (patient relevant) health outcomes requires reliable data. However, the data are often limited to a hospital setting, and hence valuable information regarding outcomes and costs is potentially overlooked. The aim of this research is to highlight the importance of this information gap by studying the association between multimorbidity and the healthcare spending among RA patients from an integrated perspective (e.g. primary care, secondary and mental care) versus a single hospital. In addition, the association between quality of life, the expenditures and multimorbidity is examined to give a comprehensive overview of the effects of a fragmented healthcare system.

## Materials and methods

### Study design and population

A retrospective cohort study design was applied to analyze the number of RA patients suffering from multimorbidity and the related healthcare expenditures. The data were obtained from a single-hospital data (Maasstad hospital) and a set of population-wide data sources (Dutch RA population). Patients who received RA care in the hospital and were at least 18 years old were included. Furthermore, inclusion was based specialist-diagnose code (0324)-101 from the Diagnosis Treatment Combinations system, which is linked to International Statistical Classification of Diseases and Related Health Problems-version 10 (ICD-10) codes M05 and M06 in the electronic health record. Diagnosis treatment combinations are a diagnostic-related group (DRG) type of system used for hospital care reimbursement [12].The final population dataset contains data from over 63,000 RA patients; RA patients who did not receive hospital care for RA in 2017, or for whom this care was not registered as such were not included. Over 2500 patients were incorporated in the single-hospital dataset.

### Data sources and collection

Two datasets were analyzed, from a single-hospital and from the Dutch RA population. The Dutch RA population data were retrieved from Statistics Netherlands, including patients from all hospitals based on the specialist diagnosis code 0324-101. The data were linked at individual level to data on outpatient medication use covered by the mandatory public health insurance scheme, and comprised information on hospital diagnosis and procedures, annual health care spending for ten categories of medical care including primary care, tertiary care, mental care [13]. Finally, the data are linked at the individual level to information on the demographics age and gender from the mandatory Municipal Registry.

Subsequently, the data were linked to the Public Health Monitor 2016 of the Community Health Services, Statistics Netherlands and the National Institute for Public Health and the Environment, which contains information on smoking, Body Mass Index (BMI), and aspects of quality of life: self-rated health and functional limitations for a sample of the population using validated scales [14]. The Health Monitor comprises of a composite questionnaire as part of a national evaluation of the health of the Dutch population and 3,421 RA patients answered this questionnaire [14].

Single-hospital data were retrieved from the outpatient rheumatology department at the Maasstad Hospital Rotterdam, a teaching hospital treating the largest population of RA patients in the Netherlands. The components of the services included all procedures provided at the Maasstad hospital, both within and outside the rheumatology department, applying DBC costs to calculate hospital expenditures. Furthermore, patients were asked to fill out the EQ-5D questionnaires every six months to assess the quality of life, however, a limited proportion of the RA patients participated in the questionnaires since the implementation of the questionnaires started in the course of the year 2017. Therefore, as a complement, the 'Patient Global' Visual Analogue Scale (PG-VAS), an element of the Disease Activity Score (DAS), is examined. Ethical approval is received through the JOINT Evaluation study (institutional code T2016-76).

### Definition of rheumatoid arthritis and classification of multimorbidity

In the EHR the ICD-10 codes M05 and M06 are registered by the physician and transformed to the Dutch reimbursement code 101. The diagnostic code is utilized to analyze the costs in both the hospital and population dataset. Multimorbidity is often used interchangeably with comorbidity, the expressions can be distinguished by the fact that in the definition of multimorbidity a dominant disease is nonexistent [15]. In this study, patients are defined as multimorbid when suffering from RA and at least one other chronic disease as defined by the Elixhauser Comorbidity index [16, 17]. Comorbidity indices are generally applied to predict the mortality, hospitalization and functioning of patients by considering the level of comorbidity [18]. We chose to use to the multimorbidities discussed by the Elixhauser index because the Elixhauser is superior to the Charlson Comorbidity Index [19, 19]. The Elixhauser index encompasses 30 different diseases in contrast to 19 morbidities concerning the Charlson Comorbidity Index, both defined by a variety of ICD-10 codes [17, 17]. To increase the generalizability of the study to other disease areas, illness specific indicators such as the International Consortium for Health Outcomes Measurement (ICHOM) standard set for inflammatory arthritis, are therefore not analyzed.

### Cost and health outcomes analysis

The results of the single-hospital analysis are compared with the Dutch RA population dataset to analyse the discrepancies when solely focusing on a hospital setting provided in one outpatient clinic. Further, the effect of multimorbidity and the quality of life of RA patients is evaluated, by the EQ-5D and self-reported measures of well-being (subsample of the Dutch RA population) [13, 22].

### Statistical analysis

Descriptive statistics are used to summarize both the hospital and population data. To examine the effects of multimorbidity on the total costs of the healthcare provided to the patients, ordinary least regressions (OLS) were conducted for both datasets separately. The linear regression model examined the association between multimorbidity and the healthcare costs (dependent variable) by adjusting for age (categorical variable) and gender (binary variable). Disease duration was unavailable in the Statistics Netherlands dataset and is therefore lacking in the population analysis. An alpha level of 5% is considered with respect to the significance levels. To determine the type of missing data, missing cases are analyzed and reported. Data analyses were conducted using the statistical software packages R and StateSE 15 and 16.

## Results

### Descriptive statistics

In Table 1, the characteristics of the RA patient populations concerning the Maasstad hospital and the (Dutch) Statistics Netherlands data are presented. Over two-thirds of the patients in both samples is female. The overall Dutch population (64.3 years, SD = 13.5) was on average older than patients in the single-hospital data (59.6, SD = 14). In the single-hospital data, for less than 1% of patients, costing data were missing.

**Table 1:** Descriptive statistics rheumatoid arthritis populations

	Single-hospital data	Dutch RA Population
Number of patients	2582	63,851
Female	1871 (72.4)	44,320 (69.4)
Age, years (mean ± SD)	59.6 (14)	64.2 (13.5)
< 50	636 (24.7)	9082 (12.2)
50–59	647 (25.1)	12,528 (19.6)
60–69	639 (24.8)	17,781 (27.8)
70–79	499 (19.3)	16,501 (25.8)
80 +	159 (6.2)	7959 (12.5)

All data are presented as total number and percentages unless stated otherwise

### Multimorbidity prevalence

The overall percentage of patients suffering from multimorbidities based on the Elixhauser is 13.1% within the RA population of the single-hospital and 23.6% in the Dutch RA population data (Table 2). The distribution of the multimorbidities shows that cardiac arrhythmias and solid tumors (excluding metastasis) were the most common diseases in both populations, but the frequency is lower in the Maasstad hospital. In the Dutch RA population, the third most frequent morbidity was hypertension (uncomplicated), while obesity and uncomplicated diabetes for single-hospital were the third most frequent in the single-hospital data. Of all conditions in the Elixhauser Index, one-third did not occur at all in the single-hospital population.

**Table 2:** Percentage patients suffering from Elixhauser Index morbidities

Elixhauser Index components	Prevalence single-hospital data (%)	Prevalence Dutch RA population (%)
Congestive heart failure	0.8	2.2
Cardiac arrhythmias	2.9	6.1
Valvular disease	1.0	1.9
Pulmonary circulation disorders	0.2	0.6
Peripheral vascular disorders	0.1	1.7
Hypertension, uncomplicated	0.0	4.6
Hypertension, complicated	0.0	0.2
Paralysis	0.0	0.3
Other, neurological disorders	0.1	1.0
Chronic pulmonary disease	0.0	1.3
Diabetes, uncomplicated	1.6	3.0
Diabetes, comp	1.4	1.4
Hypothyroidism	0.6	0.7
Renal failure	1.3	1.6
Liver disease	0.3	0.6
Peptic ulcer disease	0.2	0.1
Aids/HIV	0.0	0.0
Lymphoma	0.5	0.4
Metastatic cancer	0.1	1.1
Solid tumor, exc. metastasis	1.7	5.7
Coagulopathy	0.2	0.3
Obesity	1.6	0.7
Weight loss	1.1	0.4
Fluid and electrolyte disorders	0.0	0.2
Blood loss anemia	0.0	0.2
Deficiency anemia	0.04	1.2
Alcohol abuse	0.04	0.0
Drug abuse	0.0	0.0
Psychoses	0.0	0.0
Depression	0.0	0.1
Total	**13.1**	**23.6**

Based on Elixhauser index as described in Elixhauser et al. [18]

### Cost analysis

Hospital expenditures over a 1-year period totaled €5417 per patient in the single-hospital data and €6419 in the Dutch RA population (Table 3). Hence, spending in the single-hospital is 87% of total hospital expenditures from the population perspective and 57% of total healthcare expenditures. Furthermore, the Dutch RA population data show that hospital care spending is approximately 67% of total medical care spending for this population (€9462).

**Table 3:** Expenditures and percentage RA patients using medication and mental healthcare (extramural care)

	Single-hospital data	Dutch population data
Number of patients	2582	63,851
Outcome measures		
Healthcare expenditures (mean ± SD)	N/A	9,462 (12,352)
Hospital care expenditures (mean ± SD)	5417 (8887)	6419 (8,977)
Multimorbidity (medication use)		
Cardiovascular disease	N/A	55.6%
Diabetes	N/A	10.2%
Mental health problem	N/A	14.7%
Asthma, bronchitis, COPD	N/A	18.7%
Multimorbidity (mental healthcare use)		
Basic mental healthcare	N/A	1.2%
Specialist mental healthcare	N/A	2.4%
Mental healthcare total	N/A	3.5%
Total (unique)*	N/A	**69.3%**

*Including Elixhauser Index morbidities, N/A means no patients were registered/present within this category

When examining data on healthcare use from outside the hospital, as acquired from the Dutch RA data, the percentage patients receiving care that suggests multimorbidity is considerably higher (Table 3). Approximately half of the RA patients use medication related to cardiovascular diseases, 18% for lung diseases and approximately 15% regarding mental health issues. Furthermore, 3.5% of the Dutch RA population used mental health care, of which 2.4 percentage point used specialist, more complex mental healthcare. Taken together, almost 70% of the Dutch RA population uses medication or mental healthcare or hospital care that suggest multimorbidity.

### Association expenditures and multimorbidity

The adjusted OLS regressions regarding the Elixhauser comorbidity index show that having multimorbidities explain (R^2^) 44% of the variation in expenditures in the single-hospital data and 18% in the Dutch RA population (Table 4). Multimorbidity is associated with a larger increase in healthcare costs and is significantly higher in the Dutch RA data (€2259–€9648) than in the single-hospital data (€43–€5821), which captures only hospital care expenditures and only a subset of multimorbidities. Expensive conditions include peptic ulcer, i.e. €5821 and €7683 in single-hospital and Dutch RA population data respectively, drug abuse (€14935), depression (€13754) and psychoses (€22053), where the latter three morbidities are exclusively encountered in the population data (i.e. in other hospitals). Females experience higher expenses while the effect of age is limited or even zero after controlling for morbidities. However, the disease duration is related to increased expenditures in the single-hospital data (€1121: 2–5 years; €3043: > 5 years). The unadjusted results, i.e. without disease duration, of the regression in the single-hospital data demonstrated similar effects with respect to the significance and magnitudes in relation to the adjusted regression (see supplementary materials).

**Table 4:** OLS regression Elixhauser variables: healthcare expenditures and multimorbidity in hospital vs. Dutch RA population data

	Maasstad Hospital expenditures in euros:		Population data: Hospital care expenditures in euros (all multimorbidity indicators):		Population data: Total health care expenditures in euros (all multimorbidity indicators):	
	Coefficient	*p*-value	Coefficient	*p*-value	Coefficient	*p*-value
Intercept	3722	0.000	4977	0.000	4927	0.000
Age < 50 years 50–59 years 60–69 years 70–79 years 80 + years	ref 156 -15 -670 -1432	ref 0.677 0.968 0.100 0.019	ref -344 -279 -551 195	ref 0.011 0.038 0.000 0.303	ref -292 -510 -1435 -3133	ref 0.005 0.000 0.000 0.000
Gender (female)	625	0.037	832	0.000	166	0.02
Disease duration < 2 years 2–5 years > 5 years	ref 1121 3043	ref 0.007 0.000				
Congestive heart failure	113	0.000	5500	0.000	3360	0
Cardiac arrhythmias	72	0.000	4333	0.000	3137	0
Valvular disease	39	0.871	4108	0.000	3574	0
Pulmonary circulation disorders	558	0.000	11,424	0.000	6172	0
Peripheral vascular disorders	3410	0.148	8055	0.000	6248	0
Hypertension, uncomplicated	N/A	N/A	6663	0.000	4790	0
Hypertension, complicated	N/A	N/A	-305	0.835	438	0.638
Paralysis	N/A	N/A	13,516	0.000	7976	0
Other, neurological disorders	222	0.417	6193	0.000	2656	0
Chronic pulmonary disease	N/A	N/A	7513	0.000	4633	0
Diabetes, uncomplicated	92	0.090	6664	0.000	3962	0
Diabetes, comp	65	0.078	4585	0.000	2259	0
Hypothyroidism	226	0.287	4652	0.000	3666	0
Renal failure	157	0.000	6531	0.000	4569	0
Liver disease	35	0.528	6992	0.000	5629	0
Peptic ulcer disease	5821	0.000	10,510	0.030	7683	0.053
Aids/HIV	N/A	N/A	12,360	0.000	367	0.832
Lymphoma	43	0.019	8461	0.000	7771	0
Metastatic cancer	405	0.000	11,786	0.000	9648	0
Solid tumor, exc. metastasis	124	0.000	4244	0.000	3627	0
Coagulopathy	55	0.474	8714	0.000	6464	0
Obesity	209	0.000	5026	0.000	3726	0
Weight loss	-4	0.950	7199	0.000	4194	0
Fluid and electrolyte disorders	N/A	N/A	6687	0.000	4197	0
Blood loss anemia	N/A	N/A	5768	0.002	4160	0.001
Deficiency anemia	-284	0.580	5386	0.000	3521	0
Alcohol abuse	-1430	0.668	-727	0.725	-1140	0.483
Drug abuse	N/A	N/A	14,935	0.097	7023	0.082
Psychoses	N/A	N/A	22,053	0.002	3350	0.182
Depression	N/A	N/A	13,754	0.000	7221	0.016
Medication use						
Cardiovascular disease	N/A	N/A	1901	0.000	1118	0
Diabetes	N/A	N/A	-229	0.203	-1004	0
Mental health problem	N/A	N/A	2797	0.000	878	0
Asthma, bronchitis, COPD	N/A	N/A	1602	0.000	491	0
Mental health care use	N/A	N/A				
Basic mental health care	N/A	N/A	2311	0.000	763	0.019
Specialist mental health care	N/A	N/A	6161	0.000	301	0.184
*N* F-VALUE P-VALUE R-squared (adjusted)	2,552 74.91 0.000 0.44 (0.44)		63,851 173.42 0.000 0.2484		63,851 103.43 0.000 0.183	

N/A means no patients were registered/present within this category

### Association quality of life, expenditures and multimorbidity

Multimorbidity as measured through healthcare utilization does not capture full health differences. Table 5 shows the results from separate regressions of total healthcare expenditures on a set of quality-of-life measures corrected for age and gender in the Dutch population data. The results show that these measures capture dimensions of well-being and health-related quality-of-life that are associated with much variation in healthcare spending in the Dutch RA population. A lower quality of life results in significant enhanced total healthcare expenditures within the patient population. For instance, a one-point higher BMI is associated with €191 higher health care expenditures. And the differences in healthcare spending that are associated with difference in self-rated health and functional limitations are even larger. The magnitude of the coefficients reveals that the variation shown in this table is at least as large as the variation shown in the table with multimorbidity as measured by healthcare expenditures.

**Ta﻿ble 5:** OLS regressions of total healthcare spending quality of life measures in the Dutch RA population data

	Share of the population	Coefficient	*p*-value
Self-rated health			
Very good	0.01	(ref)	
Good	0.32	− 2412.5	0.184
Alright	0.53	2313.0	0.200
Bad	0.12	9184.2	0.000
Very bad	0.01	13,689.7	0.000
*N*	3421		
Functional limitations			
Severely limited	0.15	(ref)	
Some limitations	0.69	− 7897.7	0.000
Not limited	0.16	− 11,954.7	0.000
*N*	3404		
Smoking			
No	0.85	(ref)	
Yes	0.15	1231.1	0.042
*N*	3334		
Ever smoked			
No	0.31	(ref)	
Yes	0.53	1293.8	0.011
Yes, currently	0.16	2139.2	0.002
*N*	3185		
BMI			
	Mean (± SD)		
	26.12 (4.42)	191.9	0.000

Functional limitations are examined using the 7-item OECD limitations scale

Patients in the single-hospital data filled out the EQ-5D questionnaire on quality of life measure, corrected for age and gender. Among the 58 patients in Maasstad hospital reporting an EQ-5D score in 2017, the mean score was 0.73 (SD ± 0.19).The majority of the population scored between the 0.6 and 0.79 on the EQ-5D index, as identified by the researchers as an average to good quality of life. Poor quality of life, defined by the researchers as a EQ-5D score less than 0.40, is significantly associated with higher hospital expenditures while correcting for gender and age. The difference between experiencing an optimal quality of life (EQ-5D score equal to 1) and a health state equal to death (EQ-5D score equal to 0), in the single-hospital data lead to an average decrease of €14230 per patient (adjusted *R*^2^ = 0.14). In addition, the patients filled out the PG-VAS questionnaire (*N* = 516): average score of 40.9 (SD ± 27.9) on a scale of 0–100 (adjusted *R*^2^ = 0.02). A higher PG-VAS score, i.e. more pain, resulted an increase of €24 per point (*p* = 0.018), after correcting for age and gender.

## Discussion

The total number of patients defined as multimorbid, i.e. suffering from RA and at least one additional illness, ranges from 23.1% (Dutch RA population) to 13.1% (single-hospital) when considering the Elixhauser index and up to 69% when measuring based on outpatient medication and types of health care expenditures that suggest multimorbidity. Expenditures from a single-hospital perspective make up 84% of the population hospital expenses and 57% of the total population. Multimorbidity is associated with higher healthcare expenditures, ranging from €43–€5821 in the single-hospital data and from €2259–€9648 in the Dutch RA population data.

A possible reason for the distinction in prevalence of multimorbidities is that RA patients in the Maasstad hospital (i.e. the single-hospital site) may be treated elsewhere in the region for diseases other than RA. Since the Elixhauser indices only include a subset of diseases and sources, the overall multimorbidity rate is expected to be underestimated in the study. This is confirmed by the data on outpatient medication use and health care expenditures that we analyze, which suggest that the prevalence of e.g. chronic heart disease, lung disease and mental health problems is much higher. In clinical practice, medical staff members should be aware of the possibility of missing information concerning multimorbidities. In RA patients suffering from multimorbidities, the adherence to the treat-to-target approach, which is recommend by the American College of Rheumatology and the European Alliance of Associations for Rheumatology, multimorbidities is often suboptimal [23]. As a result, treatment responses can be lower or delayed [23].

There are large differences in the costs between the single-hospital data and the Dutch RA population data. These differences are caused because patients may receive care in other hospitals and by other types of healthcare providers. For instance, healthcare, with the exception of some psychiatric care, is not provided at general hospitals in the Netherlands and therefore costs solely become visible when examining costs outside of the hospital. As patients suffering from chronic diseases such as RA have a higher risk to develop mental disorders such as depression, looking beyond the hospital division is recommended for providers of chronic patients [24, 25].

Apart from the differences in healthcare expenditures by multimorbidity as measured by diagnoses established when using healthcare, there is also variation in quality of life in the RA patient population. Like the use-based multimorbidity measures, this variation in quality-of-life is associated with variation in health care expenditures. Quality of life measures demonstrated worsened self-rated health, loneliness and functional limitations lead to increased healthcare costs. These findings are in line with previous research regarding health-related quality of life, showing that patients suffering from arthritis have a lower reported quality of life than the general population and that there is an inverse relation between quality of life and number and multimorbidities [26, 27, 28]. Although, different quality of life measures were applied in the data sources due to the availability of the instruments, the questionnaires utilized in the study are self-rated measurements and as shown in literature, measures depict parallel examinations of quality of life [29]. Therefore, the outcomes are suitable to compare.

Analyzing the impact of multimorbidity on the expenditures and health outcomes of patients in the context of a single-hospital, only partially captures multimorbidity. The results implicate that solely a hospital perspective may not be the proper scope for treatment, interventions and evaluations from a VBHC view. Furthermore, subjective measures such as self-reported quality of life offer a broader picture than multimorbidity measures based on healthcare use and are, like the use-based measures related to higher costs in multimorbid patients.

In comparison with the study of Gunderson et al. (2021) concerning the burden of multimorbidity in RA patients, our study included a significantly larger patient population. Furthermore, the analysis performed in our study was not limited to examining the prevalence of multimorbidity within RA, but also examined the effects on costs and outcomes [1]. The incidence and prevalence of assessing multimorbidity was also based on inter alia the Elixhauser index [1]. In another study, the relationship between multimorbidity and healthcare costs in patients with musculoskeletal disorders, which also includes RA, was examined [9]. Besides impact on the direct costs as a result of hospitalization and hospital site visits, indirect costs (e.g. productivity losses) were also substantial within this patient population [9]. Although the researchers analyzed the association between healthcare costs, multimorbidity and quality of life, the focus was not specifically on RA patients,

Measuring costs from the whole spectrum of the care delivery process in the Netherlands is considered as a strength of this study. In a literature review conducted on mental health problems in patients with chronic illnesses, the authors also reported a positive association between multimorbidity and total costs [30]. The same conclusion can be drawn from a study performed in the United States, suggesting that RA suffering from depression are subject to higher healthcare utilization [31]. Similar advices and recommendations are found on the subject of multimorbidity and the proposed approach to move to a holistic practice of care delivery by incorporating elaborate data on expenditures and usage in different segments of healthcare [3, 28]. In this study, we demonstrated the effects on costs and utilization from the comprehensive perspective suggested. Additionally, addressing the challenge of multimorbidity by quantifying expenditures and the quality of life, is also viewed as an extension of the current literature, targeting for a complete picture of the multimorbidity inquiry instead of focusing on one element.

Limitations of the study include that for the Elixhauser index applied as multimorbidity proxy, a limited percentage of ICD-10 codes fall within the criteria set out in the index. Hence, the morbidities considered are not exhaustive for the whole spectrum of multimorbidity and might lead to an underestimation of the prevalence. In other studies, the reported prevalence of multimorbidity is therefore higher [1, 9]. A second limitation is that only a limited number of patients reported the EQ5D-score in 2017. The EQ-5D is an element of the Patient Reported Outcome Measures and the questionnaires have been implemented in 2017, explaining the low number of respondents. Another limitation is the fact that data from 2017 might not be generalizable to recent healthcare expenditures. On the other hand, the effect of multimorbidity on the expenditures and quality of life measures is not expected to extremely alter in a period of 4 years. Moreover, due to the variation in diagnosis registration with respect to different countries, the impact of multimorbidity on the expenditures will possibly vary per country [32]. For example, the prevalence of comorbidities in the United States in much larger than in the Netherlands. In addition, the geographic area and related socio-economic status is potentially of influence on the number of patients suffering from multimorbidities. As lower socio-economic is a predictor for the number of chronic illnesses prevalent in patients [33].

To optimize care delivery as part of the value driven care movement, insight in the variety of actors within a potential integrated practice unit (IPU) is relevant. In the end, this enables multidisciplinary steering on both the generic health outcomes and healthcare utilization (i.e. expenditures). The study addresses the knowledge gap concerning the impact of multimorbidity on the costs beyond the silos in healthcare. By analysing their relevant multimorbidities and well-being, patients can be treated as separate entities instead of a cluster of single illnesses. A previous study examining the impact of multimorbidity on payment designs, demonstrated that healthcare reforms have to advance towards a coordinated care approach to deal with the current trends of growing multimorbid populations and to diminish the burden of patients who are coping with multimorbidity [34].

Furthermore, to provide an optimal and effective treatment to patients, physicians should be aware of the comprehensive picture of morbidities of patients besides the index disease. From an extramural perspective the uptake of medication showed multimorbidity impact on healthcare utilization. These additional sources on medication provide a more extensive insight in the impact of multimorbidity in RA patients.

To demonstrate the impact of the single-hospital focus, the goal of the study was to examine the costs and quality of life of multimorbid patients from a holistic point of view. This is achieved by comparing RA patient data from a Dutch single-hospital site with the national population data on the whole spectrum of care (i.e., primary, tertiary and mental care). Concerning the transition to a value-based reimbursement system, insight in the number of multimorbid patients from a national database is also relevant as for example in bundled payments, more complex patients have to be compensated for (e.g.by receiving an additional payment). As a result of the study, the question arises why multimorbidity is associated with higher costs; i.e. due to the differences in the RA treatment resources or underlying differences between patients. Future research will focus on defining the cause of the association.

## Supplementary Information

Below is the link to the electronic supplementary material.

Supplementary file1 (DOCX 20 KB)

## Data Availability

The data that support the findings of this study concerning the Dutch RA population are not publicly available. Author PB may assist in getting access to these data. With respect to the single-hospital data, the raw data were generated at Maasstad Hospital in Rotterdam, the Netherlands. Derived data supporting the findings of this study are available from the corresponding author FK on request.
