# Supplementary material for: Multimorbidity status and annual healthcare expenditures of rheumatoid arthritis patients: a Dutch hospital-centered versus population-based comparison
Source: Rheumatol Int. 2023 Feb 10;43(6):1067–76. doi: 10.1007/s00296-023-05282-w (PMC10125938; doi:10.1007/s00296-023-05282-w)
Supplement: Supplementary file 1 — Supplementary file1 (DOCX 20 KB) [file 296_2023_5282_MOESM1_ESM.docx]

**Supplementary file A1.** OLS regression Elixhauser variables: healthcare expenditures and multimorbidity in hospital vs. Dutch RA population data (unadjusted for disease duration in the single-hospital data)

|  | Hospital care expenditures: Maasstad Hospital | | Hospital care expenditures (all multimorbidity indicators): Population data | | Total health care expenditures: all multimorbidity indicators | |
| --- | --- | --- | --- | --- | --- | --- |
|  | **Coefficient** | **p-value** | **Coefficient** | **p-value** | **Coefficient** | **p-value** |
| Intercept | 5540 | 0.000 | 4977 | 0.000 | 4927 | 0.000 |
| Age  <50 years  50-59 years  60-69 years  70-79 years  80+ years | ref  441  527  -154  -894 | ref  0.247  0.170  0.708  0.082 | ref  -344  -279  -551  195 | ref  0.011  0.038  0.000  0.303 | ref  -292  -510  -1435  -3133 | ref  0.005  0.000  0.000  0.000 |
| Gender (male) | -735 | 0.016 | 832 | 0.000 | 166 | 0.02 |
| Congestive heart failure | 116 | 0.000 | 5500 | 0.000 | 3360 | 0 |
| Cardiac arrhythmias | 73 | 0.000 | 4333 | 0.000 | 3137 | 0 |
| Valvular disease | 1 | 0.998 | 4108 | 0.000 | 3574 | 0 |
| Pulmonary circulation disorders | 529 | 0.000 | 11424 | 0.000 | 6172 | 0 |
| Peripheral vascular disorders | 3932 | 0.101 | 8055 | 0.000 | 6248 | 0 |
| Hypertension, uncomplicated | N/A | N/A | 6663 | 0.000 | 4790 | 0 |
| Hypertension, complicated | N/A | N/A | -305 | 0.835 | 438 | 0.638 |
| Paralysis | N/A | N/A | 13516 | 0.000 | 7976 | 0 |
| Other, neurological disorders | 256 | 0.356 | 6193 | 0.000 | 2656 | 0 |
| Chronic pulmonary disease | N/A | N/A | 7513 | 0.000 | 4633 | 0 |
| Diabetes, uncomplicated | 103 | 0.063 | 6664 | 0.000 | 3962 | 0 |
| Diabetes, comp | 55 | 0.143 | 4585 | 0.000 | 2259 | 0 |
| Hypothyroidism | 285 | 0.205 | 4652 | 0.000 | 3666 | 0 |
| Renal failure | 161 | 0.000 | 6531 | 0.000 | 4569 | 0 |
| Liver disease | 36 | 0.517 | 6992 | 0.000 | 5629 | 0 |
| Peptic ulcer disease | 5861 | 0.000 | 10510 | 0.030 | 7683 | 0.053 |
| Aids/HIV | N/A | N/A | 12360 | 0.000 | 367 | 0.832 |
| Lymphoma | 45 | 0.015 | 8461 | 0.000 | 7771 | 0 |
| Metastatic cancer | 386 | 0.000 | 11786 | 0.000 | 9648 | 0 |
| Solid tumor, exc. metastasis | 123 | 0.000 | 4244 | 0.000 | 3627 | 0 |
| Coagulopathy | 53 | 0.491 | 8714 | 0.000 | 6464 | 0 |
| Obesity | 210 | 0.000 | 5026 | 0.000 | 3726 | 0 |
| Weight loss | -18 | 0.779 | 7199 | 0.000 | 4194 | 0 |
| Fluid and electrolyte disorders | N/A | N/A | 6687 | 0.000 | 4197 | 0 |
| Blood loss anemia | N/A | N/A | 5768 | 0.002 | 4160 | 0.001 |
| Deficiency anemia | -173 | 0.740 | 5386 | 0.000 | 3521 | 0 |
| Alcohol abuse | -1034 | 0.0760 | -727 | 0.725 | -1140 | 0.483 |
| Drug abuse | N/A | N/A | 14935 | 0.097 | 7023 | 0.082 |
| Psychoses | N/A | N/A | 22053 | 0.002 | 3350 | 0.182 |
| Depression | N/A | N/A | 13754 | 0.000 | 7221 | 0.016 |
| Medication use |  |  |  |  |  |  |
| Cardiovascular disease |  |  | 1901 | 0.000 | 1118 | 0 |
| Diabetes |  |  | -229 | 0.203 | -1004 | 0 |
| Mental health problem |  |  | 2797 | 0.000 | 878 | 0 |
| Asthma, bronchitis, COPD |  |  | 1602 | 0.000 | 491 | 0 |
| Mental health care use |  |  |  |  |  |  |
| Basic mental health care |  |  | 2311 | 0.000 | 763 | 0.019 |
| Specialist mental health care |  |  | 6161 | 0.000 | 301 | 0.184 |
| N  F-VALUE  P-VALUE  R-squared (adjusted) | 2552  74.68  0.000  0.43 (0.42) |  | 63,851  173.42  0.000  0.2484 |  | 63,851  103.43  0.000  0.183 |  |

**Note**: N/A means no patients were registered/present within this category
